# A Review of Multimode Interference in Tapered Optical Fibers and Related Applications

**DOI:** 10.3390/s18030858

**Published:** 2018-03-14

**Authors:** Pengfei Wang, Haiyan Zhao, Xianfan Wang, Gerald Farrell, Gilberto Brambilla

**Affiliations:** 1 Key Laboratory of Optoelectronic Devices and Systems of Ministry of Education and Guangdong Province, College of Optoelectronic Engineering, Shenzhen University, Shenzhen 518060, China; 2 Key Laboratory of In-fiber Integrated Optics of the Ministry of Education, College of Science, Harbin Engineering University, Harbin 150001, China; zhaohaiyan@hrbeu.edu.cn (H.Z.); heuwangfan@hrbeu.edu.cn (X.W.); 3 Photonics Research Centre, Dublin Institute of Technology, Kevin Street, 8 Dublin, Ireland; gerald.farrell@dit.ie; 4 Optoelectronics Research Centre, University of Southampton, Southampton SO17 1BJ, UK; gb2@orc.soton.ac.uk

**Keywords:** tapered optical fiber structures, photonic applications, sensors, sensor interrogation

## Abstract

In recent years, tapered optical fibers (TOFs) have attracted increasing interest and developed into a range of devices used in many practical applications ranging from optical communication, sensing to optical manipulation and high-Q resonators. Compared with conventional optical fibers, TOFs possess a range of unique features, such as large evanescent field, strong optical confinement, mechanical flexibility and compactness. In this review, we critically summarize the multimode interference in TOFs and some of its applications with a focus on our research project undertaken at the Optoelectronics Research Centre of the University of Southampton in the United Kingdom.

## 1. Introduction

Optical fiber devices have been a significant research focus since low loss optical fibers were first proposed in the 1960s. Due to their excellent properties, which include low loss, compact structure, wide bandwidth and immunity to electromagnetic interference, optical fiber devices have been intensively exploited in many fields ranging from optical sensing to optical communication [1]. With the development of sophisticated optical fiber fabrication techniques, many types of fiber structures have been reported in the past several decades, including common fiber structures such as fiber Bragg grating (FBG), long period grating (LPG) and photonic crystal fiber (PCF), as well as more targeted structures such as tapered optical fiber (TOF), side polished fibers, interference devices and rare earth doped fibers. Fiber gratings are usually manufactured by modulating the refractive index (RI) along the fiber axis and various approaches have been developed to fabricate them, including interferometric techniques, the phase mask technique and the point-by-point technique [2,3,4]. Although fiber gratings exhibit many outstanding properties such as compact size, wavelength selectivity, and mature fabrication technique, it has inherent limitations, especially for high-precision sensing measurement. Moreover, fiber grating based sensors struggle to work at high temperatures since inscribed gratings are erased. In order to increase sensing performance, research based on a combination of fiber grating structures and fiber taper techniques has been reported [5,6]. PCF, as an emerging technology, has been intensively investigated and used in a wide range of applications, such as lasers, couplers, and sensors [7,8,9,10]. However, given its high-cost and poor compatibility to existing fiber types, PCF does present challenges for sensing applications.

Compared with the conventional fiber structures above, tapered optical fibers (TOF) can provide a number of useful features including a large evanescent field, strong mode confinement capability and small-scale diameter. Thus, TOF exhibits great potential for applications in communication systems and optical sensing, since it was reported for first time in 2003 [11]. Over the past few decades, TOFs have been investigated and widely used in many fields ranging from telecommunications to sensing. For instance, supercontinuum (SC) generation in TOFs was reported in [12]; whispering gallery mode (WGM) lasers based on Er^3+^ doped ZBLAN microspheres coupled with half-TOFs and TOFs were observed in [13] and a sensitive liquid refractive index sensor using TOF tips was demonstrated in [14]. These TOF based devices have exhibited many superior properties over conventional optical fiber structures and for this reason our group has investigated the TOF structure.

In this paper, we systematically summarize the various TOF technologies that exploit multimodal interference and also the range of sensing applications for TOF technology. In Section 2 “TOF Structures and Their Fabrication” the fundamental principles of light propagation within TOF structures and associated fabrication techniques are introduced. Section 3 “Applications of TOFs as Optical Fiber Sensors” provides an overview of sensing applications for structures in sensing for measurands such as refractive index (RI), temperature, humidity and biochemical. Finally Section 4 provides a conclusion and some comments on the future directions of TOF technology.

## 2. TOF Structures and Their Fabrication

Generally, TOF structures are fabricated by heating and tapering regular-size optical fiber [15]. The common techniques used in practical experiments include the flame brushing technique [16], the ceramic microheater brushing technique [17] and CO_2_ laser irradiation [18]. The parameters of the TOF, such as tapered waist diameter and the transition length between regular and tapered fibers, can be determined by controlling the hot region length and heating time. Here, several TOF structures addressed in our previous papers are reviewed, ranging from the STMS fiber structure, SPS fiber structure to MFC. Their fabrication techniques and theoretical analysis are also presented. It is useful to start this section on TOF structures which exploit multimodal interference with an overview of the fundamental principles of propagation in an untapered SMS structure as precursor to a similar overview of the operating principles of a tapered SMS structure, described subsequently.

### 2.1. A Singlemode-Multimode-Singlemode (SMS) Structure

**Figure 1:**
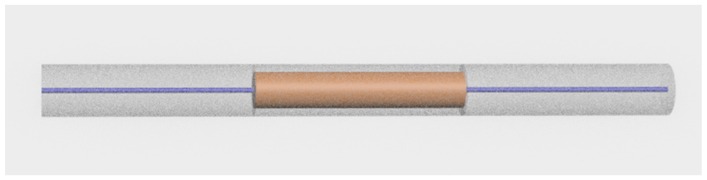
Schematic configuration of an SMS fiber structure [22]. Reproduced with permission from Xianfan Wang, Elfed Lewis, Pengfei Wang, Investigation of the Self-Imaging Position of a Singlemode-Multimode-Singlemode Optical Fiber Structure; published by Wiley Online Library, 2017.

Untapered SMS fiber structures have been intensively investigated and developed into a range of optical fiber devices, such as filters, splitters, combiners, actuators and sensors [19,20,21], due to their many merits of easy fabrication, low-cost and stable configuration. As depicted in Figure 1, an SMS fiber structure comprises a multimode fiber (MMF) sandwiched between two singlemode fibers (SMFs).

In a previous work [19], a guided-mode propagation analysis (MPA) was adopted to comprehensively investigate an SMS fiber structure. Furthermore, a comparison of the calculated self-imaging positions of an SMS fiber structure employing MPA and beam propagation method (BPM) was presented [22].

In [19] light transmission within an SMS fiber structure was firstly investigated theoretically, within which the SMF and MMF used are SMF28e (Corning) and ASF 105/125, respectively. Their parameters are follows: the SMF and MMF core/cladding diameters and RI values are 8.3/125 µm and 1.4504/1.4447, and 105/125 µm and 1.4446/1.4271, respectively. As the light propagates from the SMF to the MMF, the fundamental mode  E(r,0) is decomposed into M eigenmodes $\phi_{m}\left(r\right)$, thus:

(1) $$E\left(r,0\right)=\overset{M}{\underset{m=1}{\sum }}a_{m}\phi_{m}\left(r\right)$$

where m and $a_{m}$ respectively denote the excited mode number and excitation coefficient. Both of the parameters can be calculated from:

(2) $$a_{m}=\frac{\int_{0}^{\infty }E\left(r,0\right)\phi_{m}\left(r\right)rdr}{\int_{0}^{\infty }\phi_{m}\left(r\right)\phi_{m}\left(r\right)rdr}\;$$

(3) $$M\approx \frac{V}{\pi }=\frac{2a}{\lambda }\sqrt{n_{co}^{2}-n_{cl}^{2}}$$

where V denotes the normalized frequency parameter, a is the MMF core radius, and $n_{co}$ and $n_{cl}$ are the RI values of the MMF core and cladding, respectively.

**Figure 2:**
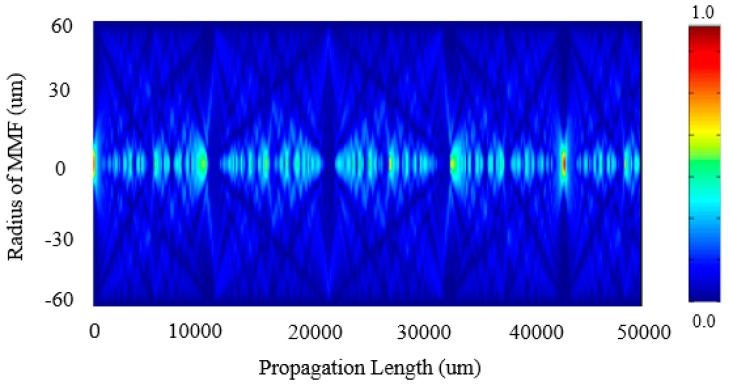
Modal field distribution along the MMF [22]. Reproduced with permission from Xianfan Wang, Elfed Lewis, Pengfei Wang, Investigation of the Self-Imaging Position of a Singlemode-Multimode-Singlemode Optical Fiber Structure; published by Wiley Online Library, 2017.

The optical amplitude distribution of the transmitted light at a propagation distance z within the MMF section can be expressed as:

(4) $$E\left(r,z\right)=\overset{M}{\underset{m=1}{\sum }}a_{m}\phi_{m}\left(r\right)\exp\left(i\beta_{m}z\right)$$

where $\beta_{m}$ represents the propagation constant of m-th mode. Based on this equation, the optical amplitude distribution pattern at a propagation distance z within the SMS fiber structure can be simulated, and the simulated result is shown in Figure 2.

When light is launched from the MMF section into the output SMF, the eigenmodes within the MMF are recoupled into the SMF fundamental mode and the relationship between the coupling loss and the MMF length can be calculated from:

(5) $$L_{s}\left(z\right)=10\log_{10}(|\overset{M}{\underset{m=1}{\sum }}a_{m}^{2}\exp\left(i\beta_{m}z\right){|}^{2})$$

**Figure 3:**
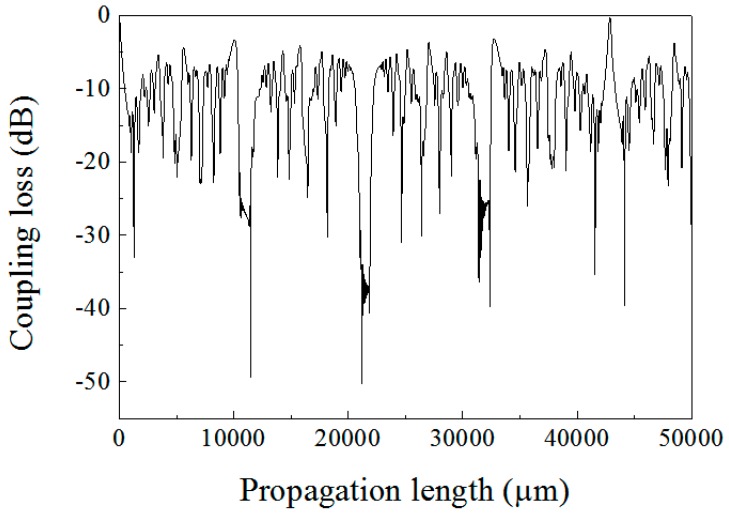
Coupling loss along the MMF.

From the above equations, the relationship between the coupling loss and the MMF length can be calculated and presented in Figure 3. The self-imaging position is denoted as the propagation distance at which both a maximum coupling efficiency and a minimum coupling loss occur. Practically edge filters and bandpass filters based on an SMS fiber structure have been proposed utilizing the spectral characteristics over a wavelength range of the multimode interference pattern at some specific MMF lengths.

### 2.2. A Singlemode-Tapered-Multimode-Singlemode (STMS) Structure and Its Fabrication

A traditional SMS fiber structure cannot be considered as a suitable candidate for many sensing applications including RI and humidity, since the thick cladding of the MMF section limits the interaction between the light propagating in the MMF and the external environment. Therefore, in order to allow an SMS fiber structure to be utilized for detecting physical quantities such as RI, humidity, gas concentration and magnetic field strength, many post-process techniques have been proposed, which include hydrofluoric acid etching, side polishing, misalignment, bend, and grating writing [4,23,24,25,26]. In [27] we were first to propose an STMS fiber structure based on a fiber tapering technique. In this sub-section, a theoretical model and an introduction to the fabrication method of the STMS fiber structure are reviewed.

**Figure 4:**
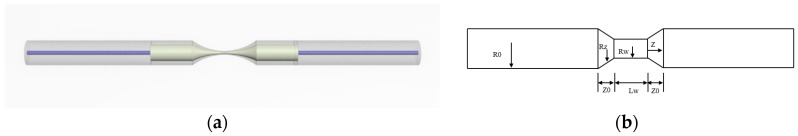
(**a**) Schematic diagram; (**b**) illustration of a STMS fiber structure [27]. Reproduced with permission from Pengfei Wang, Gilberto Brambilla, Ming Ding, Yuliya Semenova, Qiang Wu, Gerald Farrell, Investigation of Single-Mode–Multimode–Single-Mode and Single-Mode–Tapered-Multimode–Single-Mode Fiber Structures and Their Application for Refractive Index Sensing; published by OSA Publishing, 2011.

The schematic diagram of the STMS fiber structure is depicted in the Figure 4a. It consists of a section of tapered silica MMF sandwiched between two standard SMFs. Figure 4b shows the corresponding illustration of an STMS fiber structure with relevant geometrical parameters where the uniform tapered waist R(Z) and transitional length $Z_{0}$ of the tapered MMF section are respectively expressed as:

(6) $$R\left(Z\right)=R_{0}\exp\left(\frac{-Z}{L_{0}}\right)$$

(7) $$Z_{0}=L_{w}\ln\left(\frac{R_{0}}{R_{w}}\right)$$

where $R_{0}$ is the initial, radius of the untapered MMF, $R_{w}$ and $L_{w}$ are respectively the radius and length of the uniform tapered waist region and $L_{w}$ is approximated to the length of the heating region $L_{0}$.

The light propagation characteristic within the STMS fiber structure can be simulated by employing a wide-angle beam propagation method (WA-BPM) [28]. Starting with a cylindrical WA-BPM in an SMS fiber structure and given the symmetrical nature of the SMS fiber structure, the scalar wave equation can be easily calculated by adopting a cylindrical coordinate system, thus:

(8) $$R\frac{\partial^{2}E}{\partial z^{2}}+\frac{\partial^{2}E}{\partial r^{2}}+\frac{1}{r}\frac{\partial E}{\partial r}+k^{2}n^{2}\left(r,z\right)E=0$$

where k and n(r,z) respectively represent wave number in free space and the RI distribution of the MMF. Assuming a slowly varying envelop approximation for the BPM, such as $E\left(r,z\right)=\hat{E}\left(r,z\right)\exp\left(\mathrm{jk}n_{0}z\right)$, where $n_{0}$ is the reference RI, then for $\hat{E}\left(r,z\right)$, the corresponding beam propagation equation can be calculated as follows:

(9) $$\frac{\partial \hat{E}}{\partial z}=\frac{\frac{j}{2kn_{0}}P\hat{E}}{1-\frac{j}{2kn_{0}}\frac{\partial \hat{E}}{\partial z}}$$

where $P\hat{E}=\left[\frac{\partial^{2}\hat{E}}{\partial r^{2}}+\frac{1}{r}\frac{\partial \hat{E}}{\partial r}+k^{2}\left(n^{2}\left(r,z\right)-n_{0}^{2}\right)\hat{E}\right]$. With the Padé approximation [29], a Crank-Nicolson finite-difference (FD) scheme and multistep method as developed in [30,31], Equation (9) can be rewritten as:

(10) $${\hat{E}}^{l+1}=\frac{\left(1+a_{n}P\right)\left(1+a_{n-1}P\right)\cdots \left(1+a_{1}P\right)}{\left(1+a_{n}^{*}P\right)\left(1+a_{n-1}^{*}P\right)\cdots \left(1+a_{1}^{*}P\right)}{\hat{E}}^{l}$$

where $a_{i}$ and $a_{i}^{*}$ (i=1,2,⋯,n) are determined by the polynomials based on the Padé approximation. Therefore, for the i−th step, ${\hat{E}}^{l+\frac{i}{n}}$ can be solved by:

(11) $$a_{i}^{*}\eta_{-}E_{m-1}^{l+\frac{i}{n}}+\left[1+a_{i}^{*}\xi \right]E_{m}^{l+\frac{i}{n}}+a_{i}^{*}\eta +E_{m+1}^{l+\frac{i}{n}}=a_{i}\eta E_{m-1}^{l+\frac{i-1}{n}}+\left[1+a_{i}\xi \right]E_{m-1}^{l+\frac{i-1}{n}}+a_{i}\eta_{+}E_{m+1}^{l+\frac{i-1}{n}}$$

where $\eta_{\pm }=\frac{1}{\Delta r^{2}}\pm \frac{1}{2r\Delta r}$ and $\xi =\left(k^{2}\left(n^{2}-n_{0}^{2}\right)-\frac{2}{\Delta r^{2}}\right)$. At the fiber axis, r=0, the L’Hôpital rule can be applied and we have:

(12) $$\begin{array}{ll} & \left[1+a_{i}^{*}\left(k^{2}\left(n^{2}-n_{0}^{2}\right)-\frac{4}{\Delta r^{2}}\right)\right]E_{0}^{l+\frac{i}{n}}+a_{i}^{*}\frac{4}{\Delta r^{2}}E_{1}^{l+\frac{i}{n}}\\ = & \left[1+a_{i}\left(k^{2}\left(n^{2}-n_{0}^{2}\right)-\frac{4}{\Delta r^{2}}\right)\right]E_{0}^{l+\frac{i-1}{n}}+a_{i}\frac{4}{\Delta r^{2}}E_{1}^{l+\frac{i-1}{n}} \end{array}$$

Note that it is sufficient to calculate this model in half of the waveguide due to the circular symmetry of this fiber structure, which can save significant computational time and memory. The PML is also employed as the boundary condition of the computational region (r=R).

**Figure 5:**
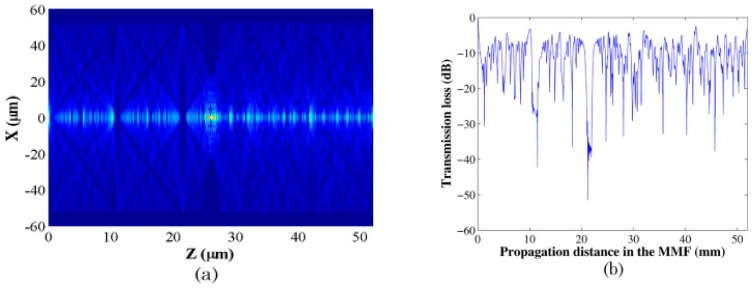
(**a**) Field distribution evolution; (**b**) coupling loss relationship along the STMS fiber structure [27]. Reproduced with permission from Pengfei Wang, Gilberto Brambilla, Ming Ding, Yuliya Semenova, Qiang Wu, Gerald Farrell, Investigation of Single-Mode–Multimode–Single-Mode and Single-Mode–Tapered-Multimode–Single-Mode Fiber Structures and Their Application for Refractive Index Sensing; published by OSA Publishing, 2011.

Finally, the coupling loss of the light field E(r,z) at a propagation distance z can be expressed as:

(13) $$L_{s}\left(z\right)=10\log_{10}\left(\frac{{\left|\int_{0}^{\infty }E\left(r,z\right)f\left(r\right)rdr\right|}^{2}}{\int_{0}^{\infty }{\left|E\left(r,z\right)\right|}^{2}rdr\int_{0}^{\infty }{\left|f\left(r\right)\right|}^{2}rdr}\right)$$

where f(r) denotes the fundamental mode of the input SMF. The output SMF is assumed to have the same fiber parameters as the input SMF. According to the equations above, the field distribution evolution along the tapered MMF section and coupling loss to the output SMF of the STMS fiber structure can be obtained, as shown in Figure 5a,b.

When the parameters of the tapered multimode fiber are substituted into the equations above, we obtain the optical amplitude distributions of the propagation fields within the STMS fiber structure and the relationship between the coupling loss to the output SMF and tapered MMF length. Comparing the simulated results of the SMS fiber structure and the STMS fiber structure, it is clear that for the STMS structures there are strong mode interferences in the tapered multimode fiber. The main reason for this is the focusing effects of the left taper transitional section. In addition, due to the tapering induced high order mode filtering effect, the mode numbers and interference intensity within the right taper transitional section are smaller than those of the SMS fiber structure.

**Figure 6:**
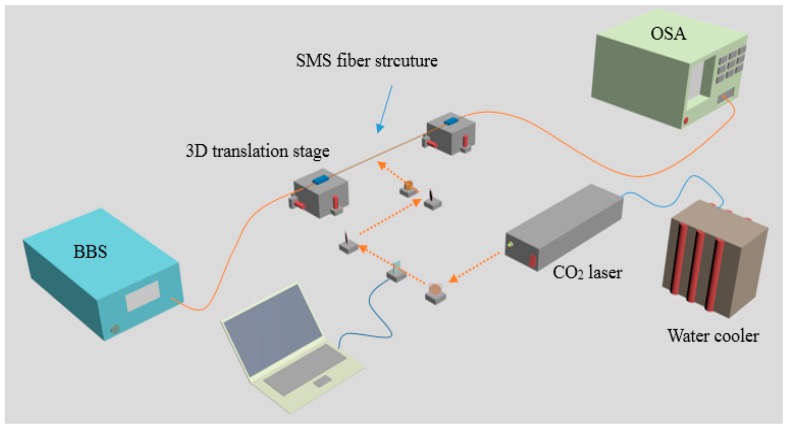
Experimental setup for fabricating an STMS fiber structure.

As regards fabrication of the STMS fiber structure, one approach is to utilise a CO_2_ laser irradiation technique [32], as shown in Figure 6. A CO_2_ laser (Model 48-2KWL Synrad, Mukilteo, WA, USA) and a ZnSe cylindrical lens with focal length of 254 ± 0.5% mm are employed in the fabrication process. It is noteworthy that the used CO_2_ laser has a maximum output power of 30 W at its working wavelength (circa 10.6 μm), and the ZnSe cylindrical lens used has focal length of 254 ± 0.5% mm.

The practical step involved firstly fabricating a traditional SMS fiber structure by splicing a specific length of uncoated MMF (ASF 105/125) with two standard SMFs with the help of a high precision cleaver and a commercial fusion splicer. Then this SMS fiber structure is fixed, taking care to avoid any bending, on two three-dimensional (3D) translation stages which can precisely move the whole fiber structure and thus adjust the heating position of this fiber structure. Meanwhile, two 3g-weights are hung on the SMF structure to ensure that the SMS fiber structure remains straight with the help of a constant applied tension. In this experiment, the position of the laser beam can be adjusted flexibly by using gold-coated mirrors on a one-dimensional (1D) motorized translation stage, while the opening and closing of its shutter can be controlled through a LabView program. When the output power of CO_2_ laser is fixed at 15 W, the CO_2_ laser beam irradiation can produce a tapered MMF.

### 2.3. Adaptation of an STMS Structure as a Probe Type Structure

**Figure 7:**
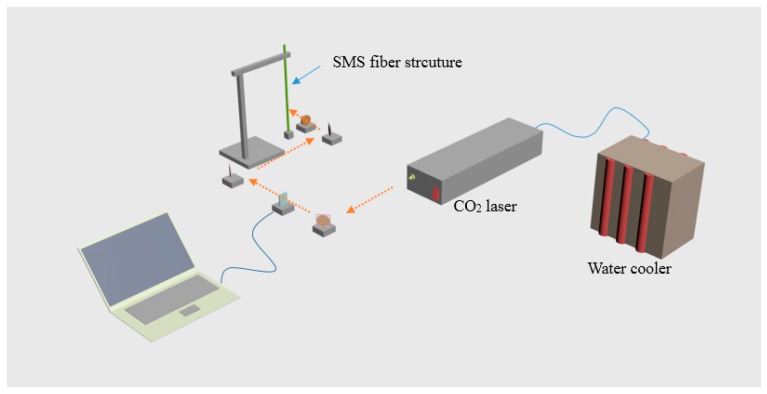
Schematic of experimental setup for fabricating a tapered MMF tip.

For some measurement environments it is beneficial to be able to utilise a sensor which has a single-ended or probe type structure which potentially can offer sensing with a high spatial resolution. As a further development of the STMS in [18], a multimode fiber tip based on the STMS fiber structure has also been proposed, to form a probe type sensor, in effect one half of a complete STMS structure which also relies on reflection from the tip. Figure 7 presents the schematic diagram of experimental setup for fabricating the tapered MMF tip structure.

**Figure 8:**
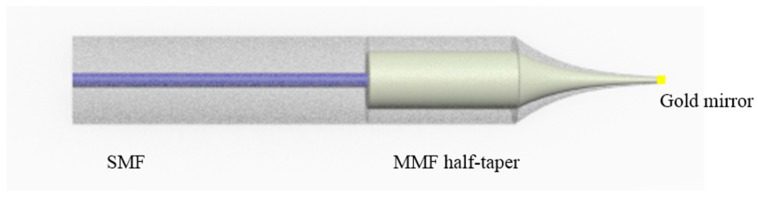
Schematic of a tapered multimode fiber tip.

**Figure 9:**
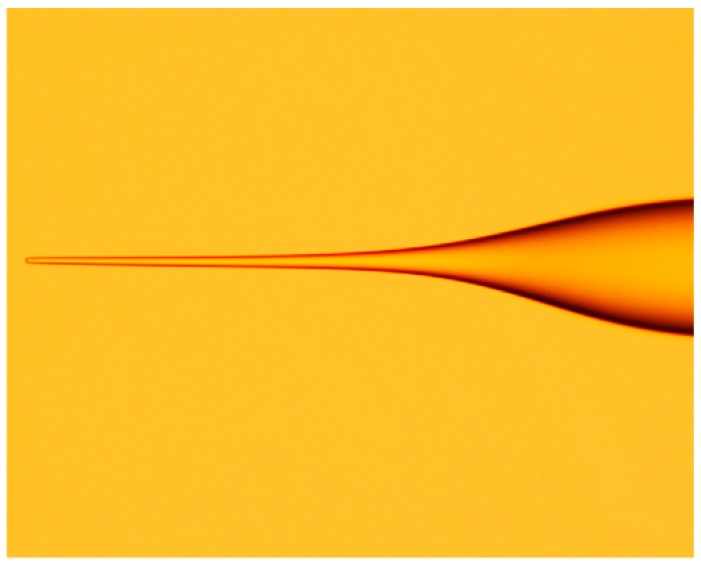
Microscope image of the fabricated tapered MMF tip [18]. Reproduced with permission from Pengfei Wang, Ming Ding, Lin Bo, Chunying Guan, Yuliya Semenova, Qiang Wu, Gerald Farrell, Gilberto Brambilla, Fiber-Tip Higherature Sensor Based on Multimode Interference; published by OSA Publishing, 2013.

The fabrication process used the same optical setup and laser parameters as the STMS structure fabrication above, but now one end of the structure was fixed vertically on a support and to the other end a constant tension was applied by a 30 g weight. With the help of the constant tension provided by the weight, when the middle of the MMF section was exposed to the CO_2_ laser beam with an average output power of 15 W, tapering occurred because of the tension applied to the fiber. Because of the tension, the fiber taper breaks uniformly at a point forming a tip. For the purpose of improving the reflection of the tip, some additional material is coated on the fiber end. A thermal evaporator is used to coat a circa 40 nm thickness of gold on the tapered MMF tip. It is worth noting that the gold film on the SMS fiber structure such as MMF long waist and conical transitional region, should be partially removed by the application of a gold-etching solvent which contains iodine and potassium. Finally, we obtain a tapered MMF tip structure with its end coated with a thin gold film, as shown in Figure 8. In addition, the microscope image of the fabricated tapered MMF tip is shown in Figure 9.

While the CO_2_ laser technique described above can be used to fabricate a tip, it does not allow the tip parameters to be determined very precisely or repeatably. Focused ion beam (FIB) technology [33,34,35] can also be used to fabricate the fiber tip. FIB technology can support the micro-operations required to form the tip with high precision, such as localized milling and metal coating deposition. In order to achieve a good manufacturing accuracy in nanoscale on fiber tips, therefore, an FIB system (Helios 600, FEI Inc., Hillsboro, OR, USA) was employed to flat cut the tapered MMF tip, which can greatly increase the reflectivity of the fiber tip. Firstly, to avoid charging during FIB milling, a gold layer with thickness of circa 50 nm was deposited on the tapered MMF tip surface by employing an electron beam evaporator. A FIB beam with a size smaller than 30 nm can be obtained through adjusting the accelerating voltage and current into optimal values (30.0 kV and 93 pA. Thus, the tapered MMF tip end shape can be controlled with precision. After FIB milling, the remained gold layer on the tapered MMF tip surface was removed totally by gold-etching solvent, which contained iodine and potassium iodine. Then, another 40 nm thickness of gold, to be used as mirror, was coated on the tapered MMF tip end to increase the reflectivity.

From a comparison of tapered MMF tip structures with and without FIB milling, we found that the FIB milling process can provide an improvement of a circa 40 dB for the reflectivity. Thus, it is demonstrated that the FIB milling technology is an appropriate approach to improve the capability of TOF-based MMF devices.

### 2.4. Periodically Tapered SMS Fiber Structures

In addition to using multimode fiber as the center section fiber, there are many other kinds of fibers used in the center section to fabricate a fiber structure similar to the SMS, such as the coreless fiber [36], the small core fiber [37], and the multi core fiber [38]. In our previous work, an SMF 28-small core singlemode fiber (SCSMF)-SMF 28 fiber structure was investigated both theoretically and experimentally [38]. Periodic tapering has also been proposed and investigated, for example a singlemode-periodically tapered small core fiber-single mode fiber structure using fiber tapering technology was investigated in [39]. In [40,41], a limited theoretical analysis of the SCSMF is presented, which assumes that there exists a interference between the core and cladding modes. However, according to the theoretical analysis in [42,43], there is no guided core mode within a SCSMF structure, which is consistent with our experimental results.

**Figure 10:**
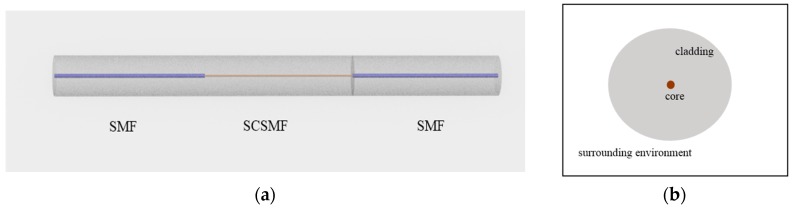
(**a**) Schematic diagram and (**b**) cross-section of the SCSMF structure.

Initially in [36], the SCSMF structure before tapering was investigated by a comprehensive theoretical analysis. To the best of our knowledge, in this a comprehensive theoretical analysis for the SCSMF structure based on an MPA was described for the first time. Figure 10a shows the schematic configuration of the SMF-SCSMF-SMF structure, and Figure 10b illustrates a cross-sectional view of it. It is clear that the SCSMF has a step index distribution and can be equivalently seen as three layers, respectively denoted as the fiber core, cladding and the surrounding environment.

When considering the circular symmetry characteristic of the input field and the SCSMF structure used, there are only zero-order azimuthal modes excited in the SCSMF of the SMF-SCSMF-SMF structure. It is assumed that the mode propagation constant and the field distribution of the SCSMF are respectively denoted as β and ψ(r) and the input field distribution can be expressed as [44,45]:

(14) $$\psi \left(r\right)=\{\begin{array}{cc} A_{0}J_{0}\left(\mu \frac{r}{a}\right) & r\leq a\\ A_{1}J_{0}\left(\mu^{\prime }\frac{r}{b}\right)+A_{2}Y_{0}\left(\mu^{\prime }\frac{r}{b}\right) & a\leq r\leq b,\;\;\;k_{0}n_{3}<\beta <k_{0}n_{2}\\ A_{3}K_{0}\left(\vartheta \frac{r}{b}\right) & r\geq b \end{array}$$

and:

(15) $$\psi \left(r\right)=\{\begin{array}{cc} A_{0}^{\prime }J_{0}\left(\mu \frac{r}{a}\right) & r\leq a\\ A_{1}^{\prime }I_{0}\left(\vartheta^{\prime }\frac{r}{b}\right)+A_{2}^{\prime }K_{0}\left(\vartheta^{\prime }\frac{r}{b}\right) & a\leq r\leq b,\;\;\;k_{0}n_{2}<\beta <k_{0}n_{1}\\ A_{3}^{\prime }K_{0}\left(\vartheta \frac{r}{b}\right) & r\geq b \end{array}$$

where $J_{0}$, $Y_{0}$, $I_{0}$, $K_{0}$ denote zero-order Bessel and modified Bessel function, a and b are the radii of fiber core and cladding, $n_{1}$, $n_{2}$ and $n_{3}$ respectively denote the RI values of the fiber core, cladding and surrounding environment. In addition, μ, $\mu^{\prime }$, ϑ, ϑ′, $A_{1}$, $A_{2}$, $A_{3}$, ${A\prime }_{1}$, ${A\prime }_{2}$, ${A\prime }_{3}$ in the above equations can be respectively defined as:

(16) $$\{\begin{array}{c} \begin{array}{c} \mu =a\sqrt{k_{0}^{2}n_{1}^{2}-\beta^{2}}\\ \mu \prime =b\sqrt{k_{0}^{2}n_{2}^{2}-\beta^{2}} \end{array}\\ \begin{array}{c} \vartheta =b\sqrt{\beta^{2}-k_{0}^{2}n_{2}^{2}}\;\\ \vartheta \prime =b\sqrt{\beta^{2}-k_{0}^{2}n_{3}^{2}} \end{array} \end{array}$$

(17) $$\{\begin{array}{c} \begin{array}{c} A_{1}=\frac{\pi A_{0}}{2}\left[\mu J_{1}\left(\mu \right)Y_{0}\left(\mu^{\prime }c\right)-\mu \prime cJ_{0}\left(\mu \right)Y_{1}\left(\mu \prime c\right)\right]\\ A_{2}=\frac{\pi A_{0}}{2}\left[\mu \prime cJ_{1}\left(\mu \prime c\right)J_{0}\left(\mu \right)-\mu J_{1}\left(\mu \right)J_{0}\left(\mu \prime c\right)\right] \end{array}\\ A_{3}=\frac{1}{K_{0}\left(\vartheta \right)}\left[A_{1}J_{0}\left(\mu^{\prime }\right)+A_{2}Y_{0}\left(\mu^{\prime }\right)\right] \end{array}$$

(18) $$\{\begin{array}{c} \begin{array}{c} {A\prime }_{1}=A\prime_{0}\left[\vartheta \prime cJ_{0}\left(\mu \right)K_{1}\left(\vartheta^{\prime }c\right)-\mu J_{1}\left(\mu \right)K_{0}\left(\vartheta \prime c\right)\right]\\ {A\prime }_{2}=A\prime_{0}\left[\vartheta \prime cJ_{0}\left(\mu \right)I_{1}\left(\vartheta^{\prime }c\right)+\mu J_{1}\left(\mu \right)I_{0}\left(\vartheta \prime c\right)\right] \end{array}\\ {A\prime }_{3}=\frac{1}{K_{0}\left(\vartheta \right)}\left[A\prime_{1}I_{0}\left(\vartheta^{\prime }\right)+A\prime_{2}K_{0}\left(\vartheta^{\prime }\right)\right] \end{array}$$

where $k_{0}=2\pi /\lambda$, c=a/b, and λ is the free space wavelength. When the field distribution of the input SMF and the field distribution within the SCSMF are assumed to be E(r,0) and ψ(r) respectively, we have:

(19) $$\psi \left(r\right)=\overset{M}{\underset{m=1}{\sum }}\psi_{m}\left(r\right)$$

(20) $$E\left(r,0\right)=\overset{M}{\underset{m=1}{\sum }}b_{m}\psi_{m}\left(r\right)$$

where E(r,0) is the eigenmode of the SMF, $\psi_{m}\left(r\right)$ is *m*-th eigenmode of the SCSMF, M is the mode number and $b_{m}$ is the excitation coefficient, which can be calculated by:

(21) $$b_{m}=\frac{\int_{0}^{\infty }E\left(r,0\right)\psi_{m}\left(r\right)rdr}{\int_{0}^{\infty }E\left(r,0\right)E\left(r,0\right)rdr}$$

When the light propagates along the SCSMF section, the field intensity distribution at distance z can be obtained from:

(22) $$E\left(r,z\right)=\overset{M}{\underset{m=1}{\sum }}b_{m}\psi_{m}\left(r\right)\exp\left(j\beta_{m}z\right)$$

where $\beta_{m}$ denotes the propagation constant of the *m*-th eigenmode. Based on the above equations, the transmission power of the output SMF can be calculated by:3

(23) $$P_{out}\left(z\right)=\frac{|\int_{0}^{\infty }E\left(r,z\right)E\left(r,0\right)rdr{|}^{2}}{\int_{0}^{\infty }{\left|E\left(r,z\right)\right|}^{2}rdr\int_{0}^{\infty }{\left|E\left(r,0\right)\right|}^{2}rdr}$$

From the above equations, it is clear that as the surrounding environment changes, the propagation constant and the excited eigenmodes in the cladding have a corresponding variation, resulting in changes of the output to the SMF.

Additionally in [38], we also investigated the influence of the core and cladding size of the SCSMF on operation and characteristics of the SMF-SCSMF-SMF structure theoretically and experimentally. We found that the cladding diameter has a much more significant influence than core diameter. The main reason for this is that since the SCSMF has a very small core, the normalized constant V ($V=\frac{2\pi a}{\lambda }\sqrt{n_{1}^{2}-n_{2}^{2}}<1$) is less than 1, which demonstrates that no core mode guides within the SCSMF.

Based on the above theoretical analysis and experimental results, it can be concluded that the SMF-SCSMF-SMF fiber structure presents an improved sensing behavior when the diameter of the SCSMF cladding becomes smaller. Traditionally, hydrofluoric acid etching cladding is an effective approach; but its induced fabrication difficulty and surface roughness are inevitable, as well as using an etching liquid is harmful to the human body and the environment [46]. Thus, a novel fiber tapering technique is adopted to replace the chemical etching method, specifically a CO_2_ laser irradiation method, which has been previously widely used to inscribe grating in conventional silica fibers [47]. Compared with the UV-laser exposure technique [3,48,49,50], the CO_2_ laser irradiation method can provide many merits of low cost, easy fabrication and flexible since there is no need for photosensitive fiber or any other pretreatment process [47,51,52,53]. When combining the fiber tapering technique and the CO_2_ laser irradiation method, a series of microtapers were fabricated to produce a periodically tapered (SPTS) fiber structure [39].

**Figure 11:**
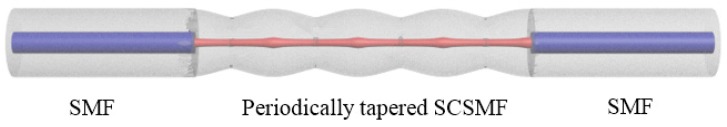
Schematic diagram of the SPTS fiber structure.

The SPTS fiber structure is composed of a periodically tapered SCSMF spliced axially with two standard SMFs, as shown schematically in Figure 11. By comparison to the SMF-SCSMF-SMF fiber structure [37], the SPTS fiber structure shows a better response to a number of physical parameters within an external environment, such as RI, because of the multiple foci effects and its induced cladding mode interference in the periodically tapered SCSMF section [54]. As illustrated in [37], the SCSMF fiber can be effectively seen as the surrounding environment as a coated cladding, because the SCSMF core has no guided mode. A WA-BPM with cylindrical coordinates using a Padé (3, 3) approximation operator and PML boundary conditions was used to simulate light transmission in the SMF-SCSMF-SMF fiber structure and SPTS fiber structure. From the calculated amplitudes of the optical fields presented in [39], it is clear that the optical power propagating through the SPTS fiber core is much lower than that in the SCSMF fiber core, because of the enhanced MMI and weak confinement of the fiber core mode.

**Figure 12:**
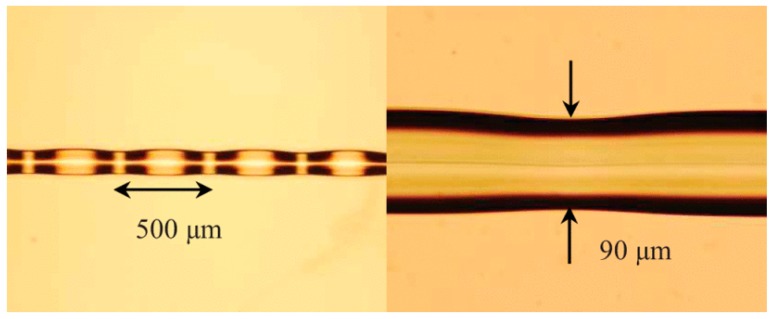
Microscope image of the fabricated SPTS [39]. Reproduced with permission from Pengfei Wang, Gilberto Brambilla, Ming Ding, Timothy Lee, Lin Bo, Yuliya Semenova, Qiang Wu, Gerald Farrell, Enhanced Refractometer Based on Periodically Tapered Small Core Singlemode Fiber; published by IEEE, 2013.

To fabricate the SPTS structure we adopted a CO_2_ laser irradiation technique, which was introduced in Section 2.2. It is noteworthy that this technique is frequently used to inscribe grating in fibers. The fabrication process for an SPTS fiber structure is similar to that for an STMS fiber structure, as depicted in Figure 7. A prefabricated SMF-SCSMF-SMF fiber structure was fixed directly on two 3D translation stages. The SCSMF section was exposed to the CO_2_ laser beam, which was moved by using gold-coated mirrors on a motorized translation stage. A LabView program was designed to control the shutter opening and closing timing. Tapering occurs when the strained SCSMF section was exposed to the CO_2_ laser beam with an output power of 10.5 W for 120 s. After that, the laser beam was moved at a distance of 400 µm along the fiber axis to heat and taper another segment of the fiber. When this taper process is repeated 20 times, a periodically tapered SCSMF structure is fabricated. It is worth noting that while the movement distance of laser beam is 400 µm, the actual distance between consecutive taper waists is circa 500 µm due to a tapering induced elongation effect. The microscope images of the obtained periodically tapered SCSMF structure is shown in Figure 12.

### 2.5. Tapered Fiber Structures Incorporating Photonic Crystal Fiber

In the last two decades, photonic crystal fiber (PCF) as a novel fiber type has attracted much attention from researchers. The cross section of a PCF consists of a solid or hollow core surrounded by a periodic arrangement of air holes [55,56,57]. These fibers provide a new approach for many applications, such as nonlinear optics, SC generation, fiber lasers, and fiber sensors [58,59,60,61,62,63]. Various optical devices based on PCF structure have been proposed, such as a liquid-crystal-filled PCF [64], LPG written PCF [65], functional material coated PCF [66], and PCF based on a side-polishing technique [9]. Among them, inscribing an LPG in PCF has been widely investigated as a basis for sensing elements since it can provide a strong wavelength dependency and a strong evanescent field, for sensor types such as a refractometer, biosensor, strain sensor and temperature sensor [67,68,69,70].

**Figure 13:**
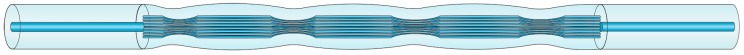
Schematic of singlemode-periodically tapered PCF-singlemode fiber structure.

**Figure 14:**
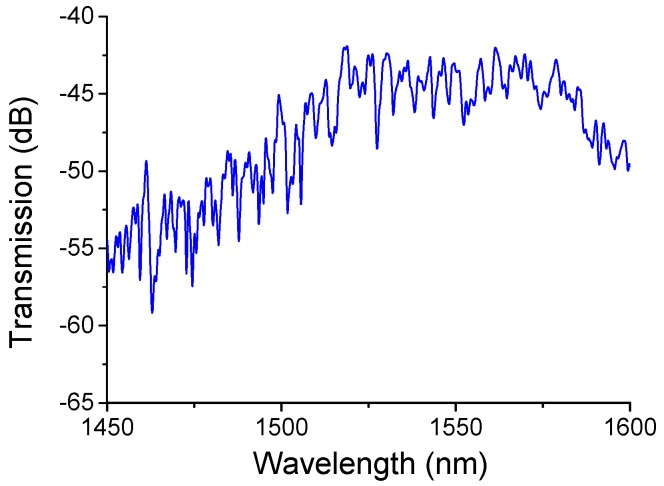
Transmission spectrum of the SPTS fiber structure when the number of periodical tapers is 40.

**Figure 15:**
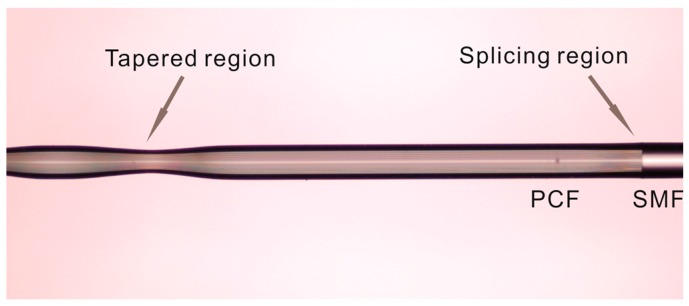
Microscope image of singlemode-periodically tapered PCF-singlemode fiber sample.

An effective approach of writing LPGs in PCF is to utilise a series of uniform microtapers, is reported in [39], is adopted to develop an SMF-periodically tapered PCF-SMF structure, as shown schematically in Figure 13. It comprises a periodically tapered PCF inserted between two SMFs. The transmission spectrum of this SMF-PCF-SMF (SPS) structure strongly depends on the spatial filtering because of the multimode interference present in the periodically tapered PCF section, as shown in Figure 14. In practice, as an initial step an SMF-PCF-SMF structure was fabricated by cutting and splicing a short length of PCF (LMA-8, NKT Photonics) with two SMFs utilizing a precise cleaver and a commercial splicer. The fusion process between the SMF and PCF is carefully executed to avoid air hole collapse [71]. The experimental setup for fabricating the SMF-periodically tapered PCF-SMF structure was similar to that used for fabricating the SPTS structure in [39], which is shown in Figure 6. In this fabrication process, a CO_2_ laser and a ZnSe cylindrical lens are employed to taper the PCF section of this fiber structure. A periodically tapered PCF section can be obtained by repeating this tapering process 40 times over. As observed previously, the actual period of the tapers was larger than the movement distance of the laser beam, due to the tapering induced elongation of the fiber. The spliced fiber joint between the singlemode fiber and the actual periodic PCF taper section is shown in Figure 15.

During the fabrication process, the transmitted spectrum of the SPS fiber structure for taper periods ranging from 0 to 40 was monitored in real time. The attenuation increased gradually for an increase in the number of tapers due to the taper transition regions that induced radiation loss. For example there is an attenuation of 10 dB associated with 10 taper periods, while the total attenuation was circa 35 dB for 40 taper periods over the wavelength range from 1450 to 1650 nm. When the number of taper periods increases, the intensity of multiple mode interferences increases correspondingly. Also the optical performance of the multiply tapered structure is determined by the physical size and the geometrical shape of the taper region. The tapering process not only induces the fiber elongation and a diameter decrease, but also causes collapse of air holes in the PCF, which in turn produces periodical effective RI modulation. Furthermore, we also investigated the transmission spectra of an SMF-periodically tapered PCF-SMF structure with different period length of 410, 430, 450, 470 µm. From these experimental results, we found no resonant interference for period lengths of 410 µm; there was a recognizable but tiny contrast interference fringe for period lengths of 430 µm, which is due to the higher loss of higher-order cladding modes. As the period lengths were increased to 450 and 470 µm, a discernable interference pattern was observed. Considering the intensity of resonant fringes, an SMF-periodically tapered PCF-SMF structure with a period length of 450 µm was thus selected for a further investigation.

**Figure 16:**
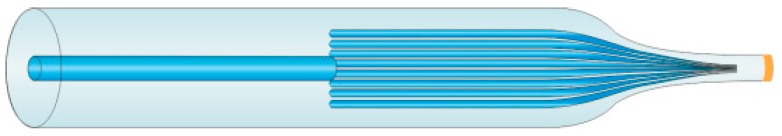
Schematic diagram of the SPST.

Although the periodically tapered fiber devices can measure the surrounding refractive index with a high resolution, the relatively large size of this fiber structure for RI sensing (usually 125 µm in diameter but several centimeters in length) restrict their sensing application in a confined space, or sensing measurements that demand a high spatial resolution. To address this issue we have developed a compact singlemode photonic crystal fiber singlemode fiber tip (SPST) structure, the schematic is shown in Figure 16.

Figure 16 presents the schematic of the SPST structure as reported in our previous work [72], and it is composed of an input/output SMF and a PCF half-taper section coated with gold film. The gold film is employed to increase the reflectivity of the tip end. Here, the SPST structure was fabricated by CO_2_ laser irradiation and is presented in Figure 7. The prefabricated singlemode-photonic crystal fiber-singlemode fiber structure is vertically fixed on a support, and a 50 g weight is hung at the other end of this fiber structure to apply a constant predictable tension. The combined effect of heating by the CO_2_ laser and the constant tension applied was to cause the glass to become molten and stretch to a point where breakage occurred, resulting in half-tapered PCF tip with a diameter of circa 3.2 µm. Furthermore, the half-tapered PCF tip was coated with a gold film of circa 40 nm thickness by utilizing a thermal evaporator to improve reflectivity. The gold film on the PCF and the conical transitional region was then partially removed using a gold-etching solvent. Finally, a thin gold film was left on the half-tapered PCF tip end. In the next stage, we expect to fabricate a SPS tip with an increased reflectivity by employing a FIB milling and gold coating process, therefore its sensing performance can be also enhanced.

### 2.6. Micro Fiber Coupler (MFC) Structure

Compared with the conventional optical fiber coupler structures, a MFC structure can effectively be used in a wider range of applications including telecommunications lasers, polarizers, and sensors [73,74,75,76,77,78,79]. For example: A dual mode fused optical fiber coupler has been fabricated for WDM transmission [79]; a non-adiabatic MFC was also used in implementing a compact, simple and high performance laser [80]; and an optical microfiber coupler based RI sensor was reported for the first time in our previously published work [81].

In this subsection structure the basic operating principles and fabrication methods for a bi-conical 2 × 2 MFC are described. Within an MFC structure, there is light interchange between the two adjacent fibers due to of the propagation constant difference between the even and odd modes [82] or symmetric and anti-symmetric modes as presented in previous literatures [83]. It is noteworthy that as the diameter of the microfiber reduces is decreased to a specific value, the transmission spectrum of the MFC structure shows a semi-periodically resonant behavior for each of the output ports. In a departure from the coupled wave equations derived in [84,85], we adopted a simple analytical method and an initial approximate theoretical analysis. When the scale of the TOF is comparable with the light wavelength, it can be approximated as a several micrometers of waveguide surrounded with an air cladding. Thus, the coupling coefficient can be calculated by [86]:

(24) $$C\left(\lambda \right)=\frac{\pi \sqrt{n_{1}^{2}-n_{2}^{2}}}{2an_{1}}e^{-2.3026\left(A+B\tau +C\tau^{2}\right)}$$

(25) $$\{\begin{array}{c} A=a_{1}+a_{2}V+a_{3}V^{2}\\ B=b_{1}+b_{2}V+b_{3}V^{2}\\ C=c_{1}+c_{2}V+c_{3}V^{2} \end{array}$$

where λ is the light wavelength, $n_{1}$ and $n_{2}$ are the RI values of the silica fiber cladding and the surrounding environment respectively, a is the radius of microfiber, τ=d∕a, the distance between the two fused microfibers is denoted as d, and the normalized frequency is calculated by V=[2πa/λ](n12−n22)12. In addition, $a_{1}=2.2926$, $a_{2}=-1.591$, $a_{3}=-0.1668$, $b_{1}=-0.3374$, $b_{2}=0.5321$, $b_{3}=-0.0066$, $c_{1}=-0.0076$, $c_{2}=-0.0028$, $c_{3}=0.0004$.

The output power of the coupled port of the MFC structure can be calculated by:

(26) $$P\left(\lambda \right)=P_{0}\cos^{2}\left(CL_{eff}\right)$$

where $P_{0}$ and P respectively denote the input power and the output power of the MFC structure, and $L_{eff}$ denotes the effective coupling length of two microfibers.

When the RI, radius and effective coupling length are determined, the calculated transmission spectra can be obtained based on the theoretical analysis above. From this, as the surrounding environment changes, a significant wavelength shift can be monitored.

**Figure 17:**
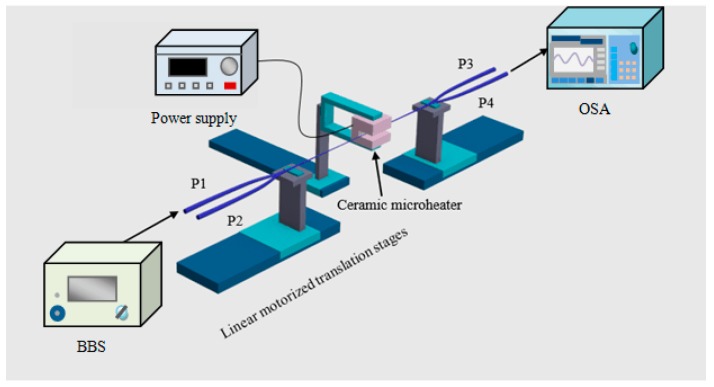
Schematic diagram of experimental setup for fabricating MFC.

Traditionally, an MFC structure is fabricated by employing a microheater brushing technique to taper and fuse two SMFs [87]. Figure 17 presents the experimental fabrication process for the MFC structure, and it mainly consists of two linear motorized translation stages and a ceramic microheater (CMH-7019, NTT-AT, Osaka, Japan), the temperature of which is regulated by adjusting the current provided by a power supply.

As an initial fabrication step, two SMFs are placed in proximity together and twisted slightly to ensure close contact. After that, the fibers are placed on two linear motorized translation stages, which can be accurately adjusted by a LABVIEW program. When the temperature of the ceramic microheater reaches about 1300 °C, the tapering and fusing begin with the silica fiber glass becoming soft. By presetting a number of control parameters, the taper diameter, length, and shape of the MFC structure can be determined. In our previous paper [81], the MFC used had a 2-mm long uniform waist region, which is fabricated by weakly fusing two microfibers with a diameter of 2.5 µm. The length of the transition region between the uniform region and input/output ports was 27 mm. The fabricated tapers should be adiabatic to support the $LP_{01}$ mode and the profile of the transitional region fell within the adiabatic regime demonstrated in [73].

**Figure 18:**
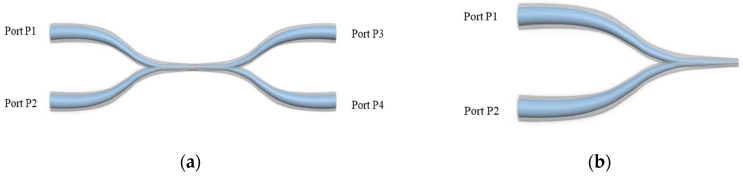
Schematic of (**a**) a MFC structure and (**b**) a MFCT structure.

A microfiber coupler tip (MFCT) was also investigated to broaden applications of MFC based devices, in relation to a high spatial resolution [88,89,90]. Figure 18 shows both an MFC and an MFCT fabricated by using a ceramic cleaver to divide the complete MFC structure into two equal parts. The fabricated MFCT comprised a uniform waist region, a conical transitional region and two input/output ports (P_1_ and P_2_). When the light is launched from port P1 to MFCT structure, and the reflected light from port P2 can be received as a result of the existed flat end surface. Moreover, the end of the MFC tip could be coated with gold film to improve its reflectivity, and therefore its sensing performance.

## 3. Applications of TOFs as Optical Fiber Sensors

A wide variety of high-performance sensors based on TOF structures have been reported, which include RI sensors [91,92,93], temperature sensors, humidity sensors [94], gas sensors [95], magnetic field sensors [96], biosensors [97], strain sensors [98] and an inclinometer [99,100,101]. In this section, we summarize several common sensing applications of the TOF structures which exploit multimodal interference for an enhanced performance. The sections ends with a short overview of the interrogation technologies for the sensors described in this section.

### 3.1. RI Sensor

RI is an important physical parameter to measure in itself but importantly measuring RI is also a foundation for sensing a variety of other measurands, for example by utlising coatings on sensors where the coating RI is influenced by the level of the desired measurand. To date many types of fiber RI sensors have been reported and developed. Traditionally, the common implementation structures include FBG [102,103], LPG [3], surface plasmons [104,105,106], SMS fiber structures [20,27,107], and TOF structures [14,95,108]. Recently, various fiber structure based RI sensors have been investigated; for example, in reference [20], the relationship between the diameter and length of MMF core within an SMS fiber structure and its RI sensing properties has been investigated; a novel RI sensor based on a hybrid structure of an SMS fiber and an FBG was demonstrated experimentally in [109]; an SMS 28-SCSMF-SMF 28 structure was demonstrated experimentally to have a maximum sensitivity of 1808 nm/RIU [38]. TOF structures have also been utilized to implement RI sensors, such as STMS fiber structures [54], SPTS fiber structures [39], SPS fiber structures [110], and MFC fiber structures [81], just to cite a few. In this section, a comprehensive review is described for the RI sensors mentioned above.

**Figure 19:**
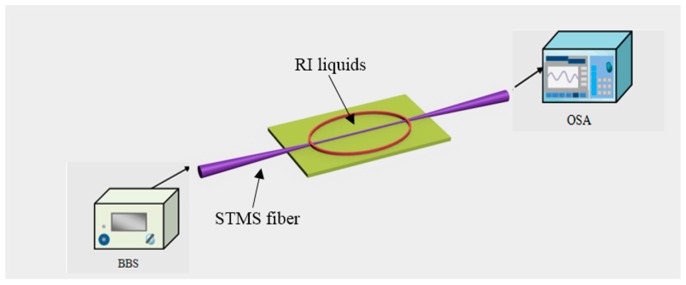
Schematic diagram of experimental setup for RI sensing.

Generally, the approach to RI measurement for different TOF structures are similar, therefore, the STMS fiber structure is taken as an example to illustrate the measurement process. Figure 19 presents the experimental setup for the RI measurement, which consists of a broadband source (BBS), an OSA, and the fabricated STMS fiber sample. The RI sensing measurement was implemented at room temperature of about 25 °C, and a series of RI liquids (1.33–1.44 with an interval of 0.01, RI error ±0.0002) was used. Broadband light with wavelength range of 1450–1650 nm was launched from a BBS, transmitted through the STMS fiber structure, and recorded by an OSA. The prepared RI liquids were placed around the STMS fiber sample by using a dropper. The taper waist region of the STMS fiber sample was immersed totally. Before dropping and measuring the next RI liquid, the remained RI liquid on the fiber was washed off by deionized water. Finally, the experimental optical spectrum shift with respect to different RI values was obtained by processing the measured data of OSA.

**Figure 20:**
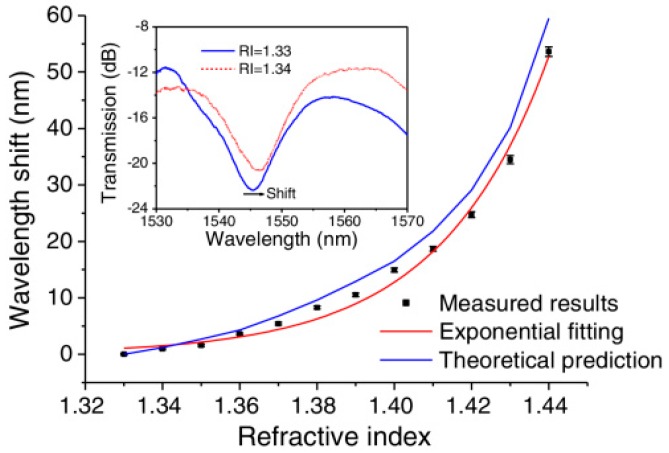
Resonance wavelength shift with RI change [54]. Reproduced with permission from Pengfei Wang, Gilberto Brambilla, Ming Ding, Yuliya Semenova, Qiang Wu, and Gerald Farrell, High-Sensitivity, Evanescent Field Refractometric Sensor Based on a Tapered, Multimode Fiber Interference; published by OSA Publishing, 2011.

The relationship between the resonance wavelength shifts and RI values was also presented in Figure 20. Over the RI range of 1.33–1.44, the RI sensor based on an STMS fiber structure has an average sensitivity of 487 nm/RIU. In addition, when the RI value is 1.44, this proposed sensor can achieve a maximum sensitivity of 1913 nm/RIU, which means that there is an RI resolution of up to 5.23 × 10^−6^ assuming the minimum wavelength shift of the OSA was 0.01 nm.

**Figure 21:**
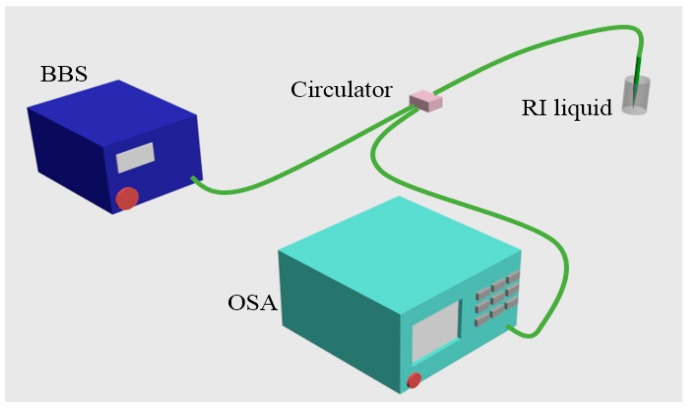
Experimental setup for RI sensing measurement.

Using a probe type sensor for RI measurement is useful for certain applications. Here, in case of trivial description, we presented the experiment process of the SPST fiber structure. As shown in Figure 21, the input/output SMF of the fabricated SPST sample was divided into two ports by adopting a circulator, which was connected to a BBS source and an OSA, respectively. The FIB-milled gold coated SPST end was inserted into a series of RI liquids of which RI values is increased from 1.33 to 1.44 with an increment of 0.01 at room temperature of about 25 °C.

**Figure 22:**
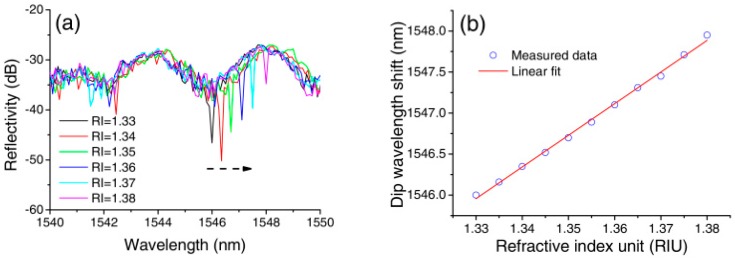
(**a**) Spectral response of the SPST sensing probe for RI increasing from 1.33 to 1.38; (**b**) linear fit of dip wavelength shifts of SPST probe [72]. Reproduced with permission from Pengfei Wang, Ming Ding, Lin Bo, Chunying Guan, Yuliya Semenova, Weimin Sun, Libo Yuan, Gilberto Brambilla, and Gerald Farrell, Photonic Crystal Fiber Half-Taper Probe Based Refractometer; published by OSA Publishing, 2014.

The ends of the SPST fiber structure are respectively connected to the BBS source and an OSA through a circulator, so as to input a broadband light and receive the reflection spectrum. From the measured results in Figure 22, the spectrum showed a redshift, and its average sensitivity is up to 39.1 nm/ RIU with RI values increment. This means the sensor could achieve a high RI resolution of 2.56 × 10^−4^ for an OSA resolvable wavelength of 0.01 nm.

**Figure 23:**
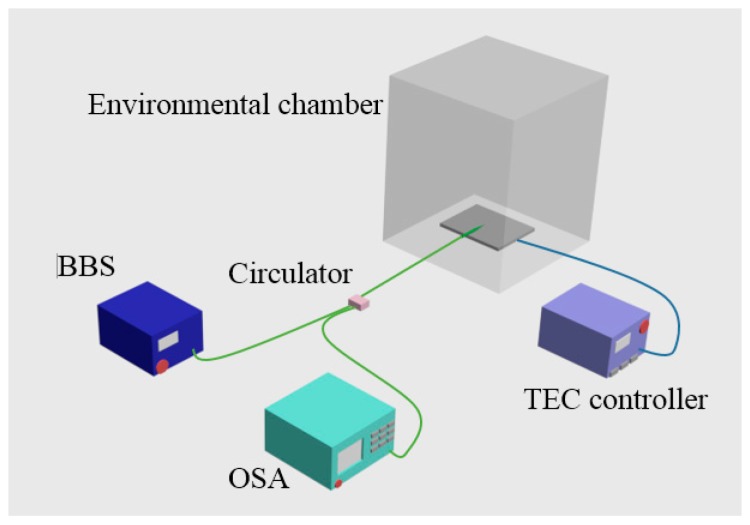
Experimental setup for studying the temperature dependence of an SPST.

Additionally the temperature dependence of the SPST based RI sensor was also investigated. Figure 23 shows the experimental setup for studying the temperature dependence of the SPST structure based RI sensor. The SPST fiber sample was fixed on a thermoelectric cooler (TEC), the temperature of which was controlled by a TEC controller. The thermometer was used to monitor the current temperature value of the TEC surface. The entire experimental setup was placed inside a controlled small environment box to improve accurate temperature regulation. The temperature dependence of the SPST fiber sample could be determined from the measured optical spectra shift over the temperature range from 20 °C to 80 °C. These experimental results exhibit that the SPST fiber structure based RI sensor has a low temperature dependence of circa 7.67 pm/°C, which is in good agreement with the intrinsic characteristic of the fused silica material.

It is also useful to systematically summarize the TOF based RI sensors proposed in our previous papers. In Table 1, a comprehensive comparison of RI range, average sensitivity, maximum sensitivity, resolution and temperature dependence are presented. It is noteworthy that MFC structure based RI sensors achieve the highest sensitivity of about 4155 nm/RIU, and it is the first time that an MFC structure with a diameter of 2.5 µm is used as a high-sensitive refractometer sensor. The most significant advantage of the SPST based RI sensor is its low temperature dependence and high spatial resolution, which offers the potential to be useful for a very broad range of applications.

### 3.2. Temperature Sensor

By comparison to traditional electronic temperature sensors, an optical fiber based temperature sensor has the merits of immunity to electromagnetic field interference and a capability to work in harsh environments and for these reasons optical fiber based temperature sensors have attracted significant research interest. A series of optical fiber based temperature sensors have been reported, most of which are based on FBGs [111,112,113], LPGs [114,115], Fabry-Perot configurations [116,117], a Sagnac interferometer [118], and an SMS fiber structure [119].

**Figure 24:**
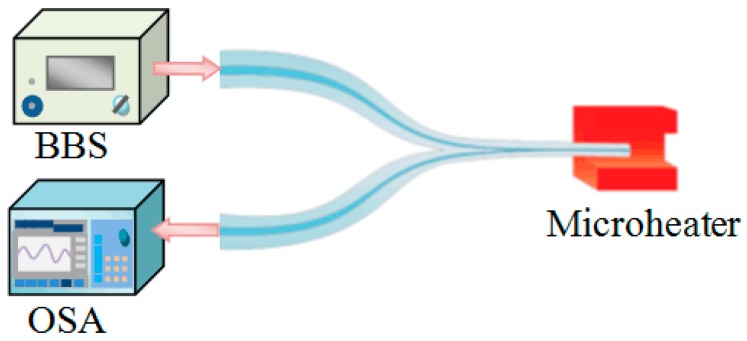
Schematic diagram of temperature measurement of an MFC tip.

In this sub-section we review various temperature sensors which utilise TOF technology, such as sensors based on an MMF tip, an MFC and MFC tip. Since the experimental setup utilized and the process for temperature sensing are similar for all of these fiber structures, only an MFC tip based temperature sensor is described here. Firstly, an MFC tip is fabricated by dividing a complete MFC structure into two equal parts, as previously illustrated in Figure 18. Two MFC tip samples, denoted as sample 1 and sample 2, were fabricated in the experiments with diameters of 12.56 µm and 4.8 µm, respectively. Considering the mechanical stability and temperature measurement range of this temperature sensor, a large diameter is preferred. This large diameter supports multiple modes resulting in multimodal interference that is strongly temperature sensitive. Figure 24 presents the experimental setup for temperature sensing measurement based on MFC tips. A BBS (Fianium Ltd., Southampton, UK) launched broadband light with wavelength range of 450–1800 nm to port P1, and the reflected light out of port P2 was received and recorded by an OSA. The microheater used (NTT-AT, Tokyo, Japan) can provide a wide experimental temperature range up to 1500 °C. Experimental results show that the measured intensity for both sample 1 and sample 2 monotonically decrease for temperature increases. The difference between two samples is that sample 1 had a lower sensitivity of 1.514 × 10^−3^ dB/°C but a wider dynamic measurement than sample 2. Considering that an achievable temperature range has a more significant meaning for temperature sensor, a high temperature sensor based on MFC tip with waist diameter of 12.56 µm was proposed. It is noteworthy that this proposed temperature sensor can work well within a wide temperature range from room temperature to 1511 °C, which is the highest temperature reported for a silica optical fiber sensor. Moreover, its response time and resolution were also measured as 16.6 ms and 0.66 °C. The sensing performance of other temperature sensors based on TOF structure (MMF tip and MFC) is included in Table 2 for comparison.

### 3.3. Humidity Sensor

Over the last few decades, accurate humidity sensing measurement has become increasingly important in numerous applications, such as industrial processing, air conditioning, civil engineering, environment controlling and many others. Optical fiber humidity sensors offer the same advantages previously mentioned elsewhere in this paper. Many types of optical fiber based humidity sensors have been reported, including a relative humidity sensor based on FBG structure coated with polymer [120], a relative humidity sensor based on LPG structure [121], a gelatin coated sub-wavelength-diameter fiber based humidity sensor with a fast response [122], and a PCF coated with PVA interferometer humidity sensor [123]. Here, a humidity sensor based on MFC structure coated with a polyethylene oxide (PEO) coating [124] is presented.

**Figure 25:**
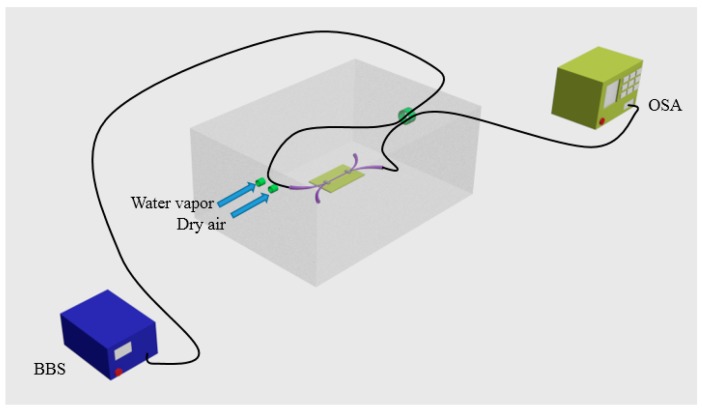
Schematic diagram of experimental setup for humidity sensing of MFC based humidity sensor.

As described in an earlier section, MFC based sensors have shown a high sensitivity to the surrounding RI variation. PEO is known as a strongly hydrophilic material and its RI value varies with the water vapor concentration [121,125,126] with the additional advantage that PEO demonstrates good adhesion to the silica of optical fiber surfaces [127]. The fabricated MFC sample with total length of 25-mm composes of a uniform 3-mm-long waist region and two 11-mm-long transition regions. The approach of the PEO solution preparation is dissolving a small amount of PEO (Sigma Aldrich, St. Louis, MO, USA) in deionized water. When the MFC was totally immersed in the PEO solution for 20 s and is taken out from the solution, the surface of MFC is coated with a thin layer of PEO film. The two MFC ends were fixed on two PEO cubes using a UV curable adhesive, which ensured a good mechanical stability. Figure 25 presents a schematic diagram of the experimental setup for humidity sensing measurements.

**Figure 26:**
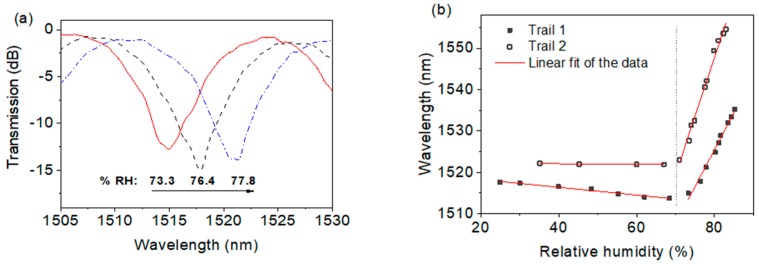
(**a**) Spectral response of the MFC humidity sensor for different humidity values; (**b**) linear fits of the two MFC humidity sensors over a humidity range of 25–85% RH [124]. Reproduced with permission from Lin Bo, Pengfei Wang, Yuliya Semenova, Gerald Farrell, Optical Microfiber Coupler Based Humidity Sensor with a Polyethylene Oxide Coating; published by Wiley Online Library, 2015.

The fabricated MFC structure coated with PEO was placed inside an environment controllable chamber (5503-00 with Package F, Electro Tech Systems, Glenside, PA, USA), of which humidity could be adjusted within a humidity range of 25–85% RH. When broadband light from a BBS (SLD 6593, Covega, MD, USA) was launched into the input port of the MFC, an OSA (Q8384, Advantest, Tokyo, Japan) was employed to record the spectral variations. Noting that the temperature inside the chamber was kept constant at 24 °C during the experiments. The RI of PEO coating decreases as relative humidity (RH) increases, causing the spectral change. The experimental results in Figure 26 show that within the humidity range of 70% RH to 85% RH, this humidity sensor based on MFC coated with PEO possesses a high sensitivity of circa 2.23 nm/% RH, as well as a good linear response.

### 3.4. Biosensor

Biosensors have important applications in biomolecule measurements such as DNA sequences and antibody and protein detection since such sensors can utilize some specific biological recognition element to investigate analytical information both quantitatively and semi quantitatively [128]. Several biosensors based on optical fiber structure have been proposed previously [129,130,131]. Recently, the development of TOF provides a potential for high-sensitivity and fast-response biosensors [132,133]. In this sub-section an MFC-structure-based label-free biosensor is presented.

**Figure 27:**
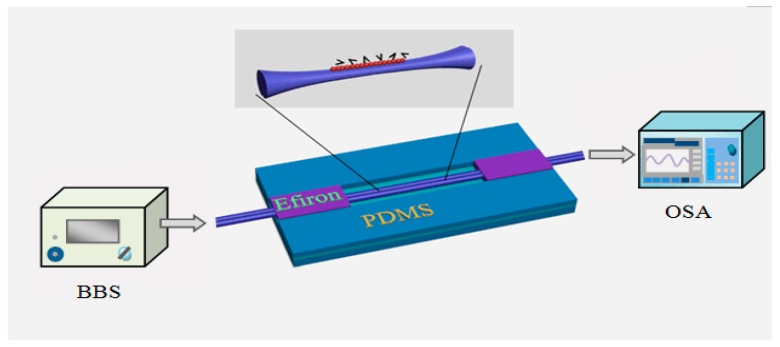
Schematic diagram of the experimental setup for MFC structure.

Firstly, a common microheater brushing technique (Figure 14) was employed to fabricate an MFC structure. The fabricated MFC structure comprised of two weakly fused microfibers with its minimum tapered waist diameter of circa 2 µm, the lengths of the uniform waist region and transition region of this MFC structure were 3-mm and 13-mm long, respectively. Based on previous work [134], this MFC is known to have an effective response to external RI variations. In order to enhance its stability, the fabricated MFC structure was covered by UV curable polymer (Efiron UVF PC363, Luvantix, Daejeon, Korea) with a low RI value. Two blocks of poltdimethylsiloxane (PDMS) were fixed in parallel on a microscope slide coated with a thin layer of the UV curable polymer by mean of which an open-top channel was constructed. The fabricated MFC was placed into the channel. Finally, the two ends of the channel is sealed with the help of s the viscous UV curable Efiron polymer, and thus the MFC structure can be immobilized. The whole experimental setup is depicted in Figure 27.

**Figure 28:**
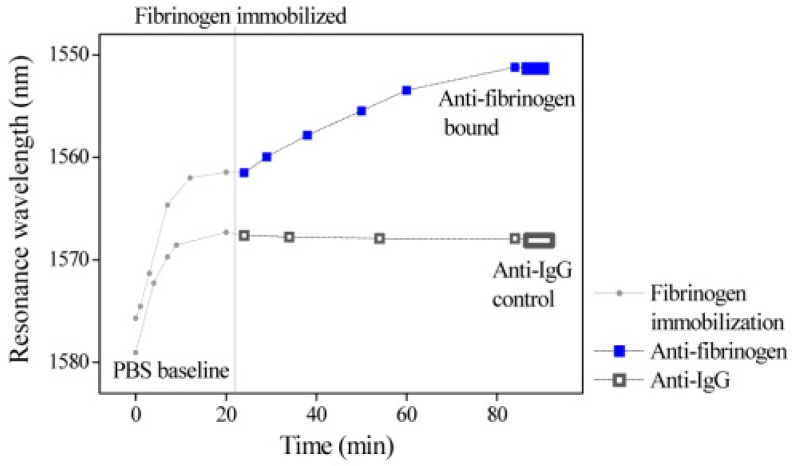
Comparison of spectral response for the anti-fibrinogen detection and anti-IgG [135]. Reproduced with permission from Lin Bo, Christy Charlton O’Mahony, Yuliya Semenova, Niamh Gilmartin, Pengfei Wang, and Gerald Farrell, Microfiber Coupler Based Label-Free Immunosensor; published by OSA Publishing, 2014.

Experimentally, a broadband light was launched from a BBS (Fiber Coupled SLD, Thorlabs, Newton, NJ, USA) to the input port of the MFC, then the output signal was detected by an OSA. Next, the fibrinogen immobilization was carried out by adopting human fibrinogen contained 95% clottable and plasminogen depleted, from Calbiochem (Merck KGaA, Darmstadt, Germany). In order to ensure that the fibrinogen molecules were totally adsorbed on the MFC surface, the MFC sensing area was covered by a fibrinogen solution in PBS (0.2 mL, 100 µg/mL) for 20 min. The inset in Figure 24 illustrates schematically the surface of the MFC with fibrinogen immobilized on it. During the entire immobilization process, the optical spectra was recorded and are shown in Figure 28. It was found that in the first five minutes, a rapid wavelength shift occurred, then the wavelength shift rate reduced and finally the wavelength shift reached a plateau within 20 min.

When the biosensor based MFC was built up, rabbit anti-fibrinogen solution (Calbiochem) in PBS buffer (0.2 mL, 100 µg/mL) and anti-IgG solution in PBS (0.2 mL, 100 µg/mL) were applied to the sensor. The experimental results exhibit that the anti-fibrinogen molecules caused a wavelength blueshift of circa 10.35 nm while the anti-IgG caused a redshift of only 0.45 nm. Therefore, it is demonstrated that this sensor can distinguish the difference between the specific anti-fibrinogen solution and the non-specific anti-IgG solution. In addition, an experiment for various concentrations of fibrinogen solution was performed. The used fibrinogen solution has a constant concentration of 100 µg/mL, while the concentration of anti-fibrinogen solution was increased from 25 µg/mL to 100 µg/mL. Based on experimental results in Figure 28, we can find that the sensor output was proportional to the concentration of the anti-fibrinogen. It is noteworthy that this is the first time a label-free MFC based biosensor using a fibrinogen and anti-fibrinogen pair has been demonstrated experimentally.

### 3.5. Sensor Interrogation

Finally and briefly, while not in itself a sensing type, an important underpinning part of a complete optical fiber based sensing system is the sensor interrogation sub-system. The most common techniques used to interrogate optical sensors involve detection of the sensor’s response to a measurand in one of three different domains: Wavelength, intensity or polarization. The nature of the sensor will determine the domain used by the interrogation system.

Measurements in the intensity domain, for example for sensors based on simple tapered optical fibers, offer the advantages of simplicity but can suffer from an undesirable dependence on fluctuations in the optical source power. Wavelength domain interrogation, for example for MFC based sensors, can be less dependent on optical source power fluctuations and can be used where multiple sensors using wavelength division multiplexing are used, but in general the interrogation system is more expensive to implement than that in the intensity domain. The most common approaches to wavelength measurement include passive ratiometric wavelength measurement schemes and active wavelength scanning schemes [136,137,138,139].

## 4. Conclusions and Outlook

This paper has reviewed several kinds of common TOF structures including STMS, SPST and MFC structures, including their theoretical simulation, fabrication methods and characteristics. The applications of sensing for TOFs were reviewed as well. TOF structures can be configured as sensors for a variety of measurands and in this paper a number of examples have been consider involving sensors for RI, temperature humidity, bio-sensing. Sensing of other parameters using TOF structures is also possible, with demonstrations for example of sensors for volatile organic compounds (VOCs), hydrogen gas concentration and mechanical pressure. Many of these sensors depend on novel coatings which allow the inherent RI sensitivity of the underlying TOF structure to be exploited. Surface Plasmon Resonance (SPR) has also been combined with TOF technology as an approach to increasing sensor sensitivity.

However, compared with other fiber structures such as fiber Bragg gratings and Mach-Zehnder interferometers, TOF is at a less well developed stage. There are a number of limitations to overcome in the near future, for example, a suitable packaging method for TOF devices is needed to offer mechanical protection for the fragile tapered fiber structure and allow protection from dust and other contaminants as they can cause long-term deterioration in the performance of TOF based devices. In addition, there is a need for improvement in fabrication accuracy and repeatability in order to allow TOF based sensors to be commercially developed. If these challenges are successfully addressed then it is possible that a range of commercialized TOF based devices can be developed and used in practical applications.

## Figures and Tables

**Table 1:** Comparison of sensing parameters for different RI sensors proposed in our previous works.

Fiber Structure	RI Range	Average Sensitivity	Maximum Sensitivity	Resolution	Temperature Dependence
STMS	1.33–1.44	487 nm/RIU	1913 nm/RIU	5.23 × 10^−6^	
SPTS	1.33–1.38	226.6 nm/RIU	383 nm/RIU	4.41 × 10^−5^	0.13 nm/°C
SPS	1.33–1.38	222 nm/RIU	232 nm/RIU	3.24 × 10^−5^	8.4 pm/°C
SPST	1.33–1.38	39.1 nm/RIU		2.56 × 10^−4^	7.67 pm/°C
FIB-milled gold coated SMST	1.33–1.40	265 nm/RIU		3.77 × 10^−5^	
MFC	1.3340–1.3800	2723 nm/RIU	4155 nm/RIU	3.67 × 10^−5^	

**Table 2:** Comparison of sensing parameters for different temperature sensors proposed previously.

Fiber Structure	Temperature Range	Average Sensitivity	Resolution	Response
MMF tip	20–1089 °C	11.4 pm/°C	0.877 °C	
MFC tip	85–1511 °C	1.514 × 10^−3^ dB/°C	0.66 °C	16.6 ms
MFC	701–1029 °C	36.59 pm/°C		
